# Testing the Effect of Metabolic Rate on DNA Variability at the Intra-Specific Level

**DOI:** 10.1371/journal.pone.0009686

**Published:** 2010-03-15

**Authors:** Angela McGaughran, Barbara R. Holland

**Affiliations:** 1 Allan Wilson Centre for Molecular Ecology and Evolution, Massey University, Palmerston North, New Zealand; 2 Institute of Fundamental Sciences, Massey University, Palmerston North, New Zealand; American Museum of Natural History, United States of America

## Abstract

We tested the metabolic rate hypothesis (whereby rates of mtDNA evolution are postulated to be mediated primarily by mutagenic by-products of respiration) by examining whether mass-specific metabolic rate was correlated with root-to-tip distance on a set of mtDNA trees for the springtail *Cryptopygus antarcticus travei* from sub-Antarctic Marion Island.

Using Bayesian analyses and a novel application of the comparative phylogenetic method, we did not find significant evidence that contemporary metabolic rates directly correlate with mutation rate (i.e., root-to-tip distance) once the underlying phylogeny is taken into account. However, we did find significant evidence that metabolic rate is dependent on the underlying mtDNA tree, or in other words, lineages with related mtDNA also have similar metabolic rates.

We anticipate that future analyses which apply this methodology to datasets with longer sequences, more taxa, or greater variability will have more power to detect a significant direct correlation between metabolic rate and mutation rate. We conclude with suggestions for future analyses that would extend the preliminary approach applied here, in particular highlighting ways to tease apart oxidative stress effects from the effects of population size and/or selection coefficients operating on the molecular evolutionary rate.

## Introduction

The rate of molecular evolution is known to vary in three predominant ways (changes in: mutation rate, population size, and selection coefficients; [1]), but in practice, the specific causes of rate variation are difficult to isolate [2], [3]. Recent interest has focused on the contribution of nucleotide mutation rate to the rate of molecular evolution. While general trends of this rate variation can be attributed to differences in repair equipment among taxa [1], multiple variables are expected to affect mutation rate itself.

Mutations arise through unrepaired errors accrued during DNA replication and other damage-causing processes. However, the mutation rate may also be influenced by the life history of a species [1], [4]. Thus, factors including body size, generation time, and metabolic rate may play important roles in determining the evolutionary rate of taxa through their effects on the mutation rate. Recently, mutation rates have been analysed indirectly using these biological variables with the intention of developing an all-encompassing theory to describe the patterns and processes of evolutionary rates for a range of taxa [2], [5]–[11]. According to proposed theories, animal taxa with large body sizes, long generation times, and low mass-specific metabolic rates should have a slower mutation rate [12]–[15]. This is consistent with observations that ectotherms have lower evolutionary rates than endotherms and that small vertebrates with high metabolic rates have higher substitution rates than large vertebrates with lower metabolic rates [1].

Metabolic rate and generation time (both correlated with body size) may affect mutation rates by altering the mean residence times of nucleotides, such that these would tend to be shorter in small, short-lived and metabolically active species [16]. In particular, normal cellular metabolism is well established as a source of reactive oxygen radicals — harmful by-products that can account for the background levels of oxidative DNA damage detected in normal tissue [17]. In healthy organisms a small but significant part of respiratory activity generates such radicals (e.g. hydroxide: OH^−^), which are capable of modifying several types of macromolecules, including DNA [17]. Antioxidants eliminate many of these radicals; however the remaining fraction can cause significant damage. This is generally expected to occur near the sites of radical generation, since the most reactive radicals are poor diffusers [13]. Thus mtDNA (the site of respiration) is likely to be a prime target for oxygen-radical caused damage, and indeed it has a higher rate of molecular evolution than nuclear genes in animals [1].

Mitochondria are able to repair at least five different types of DNA damage [13]. However, the repair of mtDNA oxidative damage has been reported as a relatively error-prone process [18]. In relation to a ‘metabolic rate hypothesis’, rates of mtDNA evolution are postulated to be affected primarily by by-products of respiration (e.g. oxygen radicals) which can cause mutations in DNA. Thus, if reactive oxygen radicals have a mutagenic effect on DNA, then taxa with higher metabolic rates should generate higher concentrations of mutagens and sustain more DNA damage. Indeed, empirical studies have demonstrated that species with higher metabolic rates experience higher rates of reactive oxygen radical production [19] and higher rates of oxidative DNA damage [20]–[22].

Interest in metabolic rate for its influence on nucleotide rate variation among populations was stimulated by Martin and Palumbi [5] and Rand [22] who documented effects of body size, temperature and correlated variables, including generation time and metabolic rate, on DNA substitution rate across various animal species. More recent work has supported these findings [10], [11]. The mass-specific metabolic rate model of Gillooly et al. [7] used thermodynamic equations to relate temperature and body size to metabolic rate and Gillooly et al. [8] demonstrated that body mass, temperature and metabolic rate explain a significant fraction of the variance in nucleotide substitution rates in a broad sample of organisms. Most recently, Gillooly et al. [9] showed that rates of protein evolution are largely controlled by mutation rates, which in turn are strongly influenced by individual metabolic rate.

Several studies have addressed this question through empirical work [5], [6], [12], [22]–[25]. However, studies of this type have run into inherent difficulties because the effects of different variables on evolutionary rates are hard to tease apart. Divergent groups of taxa usually differ in many respects (e.g. nucleotide generation time, G + C content, various life history traits), making it difficult to isolate single factors acting on DNA evolution. Therefore investigations of rate heterogeneity at an intra-specific level may be helpful, through their avoidance of these particular confounding factors [26].

Indeed, few studies to date have examined intraspecific levels of rate heterogeneity. Notwithstanding the difficulties of teasing apart population size and selection effects on rates of evolution (see above), this is most likely due to the relatively small number of mutational events that occur between closely related samples, making it difficult to achieve a statistically significant number of nucleotide mutations between sequences. Choice of a relatively fast-evolving mtDNA gene may go some way towards overcoming this.

Felsenstein [27] pointed out that comparative studies must account for the hierarchically structured phylogeny that underlies all species when assessing whether one physiological variable is correlated with another. This is because treating species as the units of analysis in a comparative study assumes that the traits under investigation evolved independently in each lineage. Owing to the phylogenetic structure of the data, however, the species will share some portion of the path leading from the root to the tips of a phylogenetic tree, and for closely related species, this may be most of the path. As a consequence, if taxa with a certain physiological trait are all closely related, they will tend to have a low genetic distance to each other regardless of their trait status [28]. Thus, ignoring the underlying coalescent history of taxa will almost certainly bias estimates of any correlation. Fortunately, there are techniques available which can account for the underlying phylogeny when examining hypotheses about trait data [28].

Here, we attempt to use such a method to examine the relationship between metabolic rate and mtDNA (*cox1*) mutation rate. We use mtDNA because, although data on mtDNA repair in non-model organisms are scarce, there is evidence that mtDNA repair efficiency varies in natural populations and may thus be influenced by natural selection [25], [29]–[33]. It is also recognised that molecular evolution can be highly variable within and among taxonomic groups, at least for mtDNA [34]. Further, evolutionary lability in metabolic traits including metabolic rate (and by inference, DNA damage rate) mean that variation among individuals will exist at the intra-specific level. Collectively, variation in mtDNA may therefore be used to explore the intraspecific relationships between metabolic rate and the rate of DNA mutation (as opposed to the rate of nucleotide substitution, which is a population-level measurement involving the incorporation of somatic mutations into the germ line).

We explore this by making use of an existing metabolic rate and mtDNA (*cox1*) dataset for the insect-like (arthropod) springtail *Cryptopygus antarcticus travei* Déharveng, 1981 (Collembola, Isotomidae) from sub-Antarctic Marion Island. This existing dataset (which forms part of a broader project; see [35], [36]) demonstrates both genetic and metabolic differentiation among these springtail populations [37], hence we use it to examine the relationship between metabolic rate and mtDNA (*cox1*) mutation rate. Using data on relative intra-specific genetic divergence, we test the hypotheses, that: (1) mass-specific metabolic rate (the trait) is positively correlated with mtDNA genetic distance (i.e., mutation rate) among *C. a. travei* individuals; and (2) this trait is dependent on the underlying mtDNA tree.

## Methods

### Location and Sample Collection

**Figure 1:**
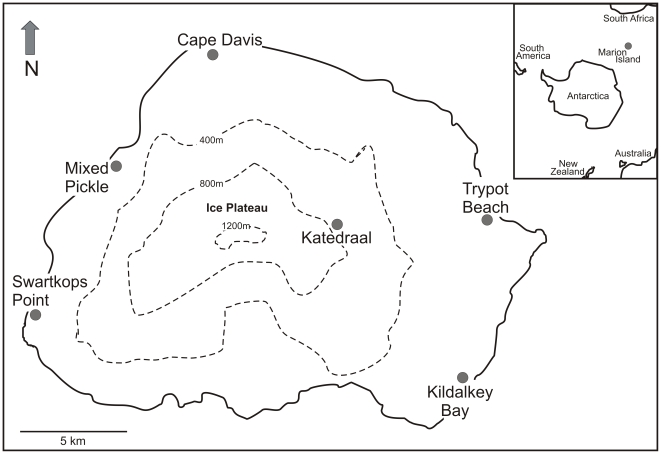
Geographic locations on Marion Island referred to in the text. Inset: Marion Island's location in the Indian Ocean.

Marion Island (46°54′S, 37°55′E) forms part of an isolated archipelago in the Indian Ocean sector of the Southern Ocean. Adult specimens of the springtail *C. a. travei* were collected from six locations across the island (Figure 1) during a 3-week period in April 2007. Extreme care was taken to ensure all samples approximated a similar body mass. We make the assumption that factors such as generation time and longevity did not differ greatly among samples/populations; this was not possible to measure, but it seems reasonable. After collection, samples were kept outside the laboratory for a minimum of one day and a maximum of three days in order to maintain near-natural field conditions. Individuals were then moved to plastic vials containing a moist Plaster-of-Paris base and moss shoots as a food source, and stored at 10°±0.5°C in a Sanyo MIR incubator (Sanyo E & E Europe, Loughborough, UK) (12∶12 L∶D) for 24 h prior to metabolic rate measurement.

### Metabolic Rate Measurements

The method employed to measure rates of oxygen consumption followed that of [36]. In brief, a fiber-optic oxygen sensing system (Ocean Optics Inc., Florida, USA) was used to monitor oxygen partial pressure (*pO_2_*) over time for individual animals in a closed respirometry system calibrated by the manufacturer for multiple temperatures and oxygen percentages.

Following calibration, individual animals that had been starved for 24 h were placed in a custom-made 40 µl chamber [36], into which the oxygen probe was inserted. During a 3 h period, temperature and *pO_2_* in the chamber were recorded continuously using oxygen sensing software (OOISensor ver. 1.05, OceanOptics Inc., USA). Temperature during runs was held at 10°±0.1°C using a Sable Systems PTC-1 cabinet (Sable Systems, Las Vegas, USA). This measurement temperature was slightly higher than the average summer microhabitat temperature later measured at Marion Island [36], however, was selected primarily for comparability to existing metabolic rate work on springtails in continental Antarctica [36].

Upon completion of a run, partial pressure profiles were used to calculate oxygen consumption rates for each individual, and an estimate of individual animal mass was used to express corresponding oxygen consumption rates on a mass-specific basis (see [36] for further information, including quality control). Photographs of individual springtails were measured using image analysis software (Leica Application Suite, Leica Microsystems, South Africa) and mass was estimated using the relationship: W = 6.1894L^3.119^×10^−9^ (after [38]), where W =  mass (µg), L =  length (µm), as modelled for the nominate subspecies *C. a. antarcticus* on maritime Antarctic Signy Island.

### DNA Extraction, Amplification and Sequencing

Mitochondrial DNA cytochrome *c* oxidase I (*cox1*) sequences were obtained from all individuals for which a metabolic rate was measured (n = 45). Extraction, thermal cycling and sequencing conditions are outlined in [35].

### Haplotype Network Analysis

**Figure 2:**
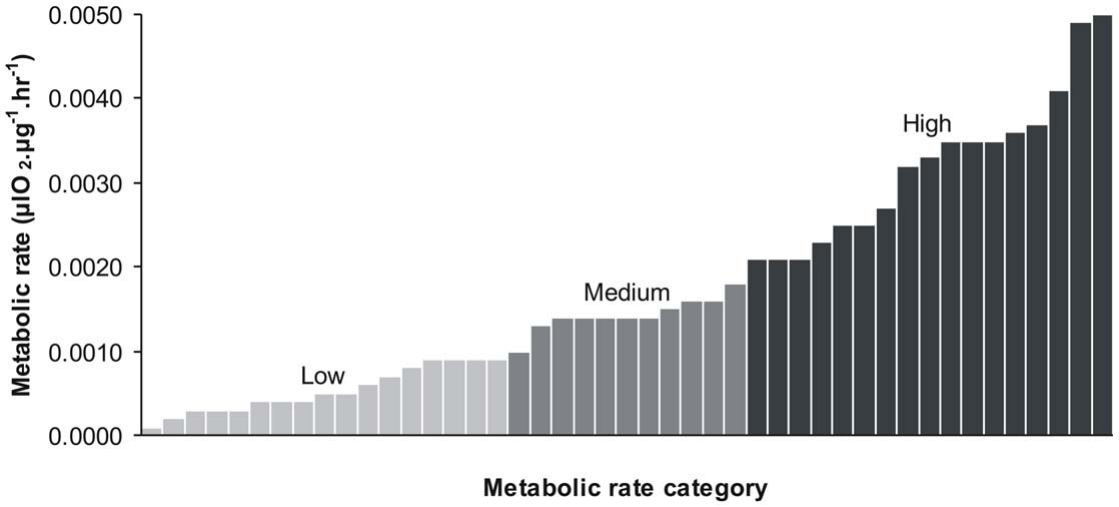
Bar-graph of metabolic rate categories. Metabolic rate data is grouped into three roughly equal-sized categories corresponding to ‘low’ (<0.0010 µlO_2_.µg^−1^.hr^−1^; n = 17; light grey bars), ‘medium’ (0.0010≤*x*≤0.0020 µlO_2_.µg^−1^.hr^−1^; n = 11; medium grey bars), and ‘high’ (>0.0020 µlO_2_.µg^−1^.hr^−1^; n = 17; dark grey bars).

Tcs ver. 1.21 [39] was used to estimate a haplotype network using the statistical parsimony algorithm of [40] and a connection limit of 95%. Metabolic rate data was grouped into three roughly equal-sized categories corresponding to ‘low’ (<0.0010 µlO_2_.µg^−1^.hr^−1^; n = 17), ‘medium’ (0.0010≤*x*≤0.0020 µlO_2_.µg^−1^.hr^−1^; n = 11), and ‘high’ (>0.0020 µlO_2_.µg^−1^.hr^−1^; n = 17) (see Figure 2) and mapped onto this network to give a graphical representation of any relationship between mutation rate and metabolic rate. These groupings are for visualisation purposes only, and were not used in the statistical analyses.

### Combined Metabolic Rate and DNA Mutation Rate Analyses

To explore whether metabolic rate is correlated with evolutionary rate (distance from the ‘ancestral’ haplotype), we took two approaches. In doing so, we rely on four assumptions: (1) that different haplotypes experience similar diurnal and annual temperature regimes; (2) that the temperature-dependence of metabolic rate is constant among haplotypes; (3) that seasonal variation in respiratory acclimation is constant among haplotypes; and (4) that distinct populations are not so distinct as to preclude the grouping together of metabolic rate measurements (since individuals with metabolic rates falling into all of our pre-defined ‘low’, ‘medium’, and ‘high’ categories are present in each of the populations, we feel this last assumption is justified). We also make use of the assumption commonly employed in studies of population genetics – that the haplotype which is most frequent and geographically widespread corresponds to the ‘ancestral’ haplotype [see 41]. We recognise that violation of any of these assumptions may violate our analytical approaches.

Of our two approaches, the first was based on the haplotype network directly; we calculated two test statistics that were designed to measure: (1) if similar haplotypes had similar metabolic rates, and (2) if haplotypes that were further from the putative root of the network had higher metabolic rates. The significance of these two test statistics was assessed using a randomisation test. The second approach was to perform correlation analysis on a set of trees generated using Bayesian analyses in the program BayesTraits ver. 1.0 ([42]; available from www.evolution.rdg.ac.uk). These approaches are outlined in more detail below.

#### Randomisation test analyses

The first test statistic, *T*_1_, was the sum, over all pairs of haplotypes whose distance in the haplotype network was less than *k*, of the absolute value of the difference in metabolic rate:
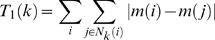
where an individual *j* is in the neighbourhood *N_k_*(*i*) of an individual *i* if the distance in the haplotype network between *i* and *j* is less than *k*, and *m*(*i*) is the metabolic rate of individual *i*.

The second test statistic, *T*_2_, was the sum, over all directed edges (*u*,*v*) in the haplotype network, of the average metabolic rate of individuals at node *v* minus the average metabolic rate of all individuals at node *u*.

The null distribution of each of these test statistics was determined by reassigning the metabolic rates to individuals at random without replacement (i.e., shuffling the metabolic rates) 1000 times, each time recalculating the test statistic. The most prevalent haplotype was used as the putative root of the network, and all edges were directed away from the root. For each test statistic we report a *p*-value, which is the proportion of the 1000 randomisations that the value of the test statistic was higher than for the real (un-shuffled) metabolic rates.

#### BayesTraits (correlation) analyses

BayesTraits implements two models of continuous trait evolution: the standard constant-variance random walk model (Model A), in which the given trait evolves randomly (i.e., with no overall tendency to increase or decrease) along a phylogenetic tree; and a directional random walk model (Model B), in which the trait tends to either increase or decrease along the tree, leading to the expectation that mutational steps from the root will be correlated with the trait value [42].

In the standard BayesTraits approach Model A and Model B are compared in order to test the hypothesis that tips further from the root have different average trait values from tips nearer the root due to directional selection [42]. Here, we use BayesTraits in a novel way, however, the hypotheses tested produce the same patterns: if there is a causal link between mass-specific metabolic rate and mutation rate (as suggested by the lines of reasoning given in the Introduction) then metabolic rates will be higher for tips that are further from the presumed root of the tree (i.e., that have higher mutation rates) (Model B pattern), whereas if there is no direct correlation between metabolic rate and mutation rate there should be no trend of higher metabolic rates for tips that are further from the presumed root of the tree (Model A pattern).

In the analyses presented here, we determine significance of results based on the use of Bayes Factors as described in the BayesTraits manual ([42]; www.evolution.rdg.ac.uk). The use of Bayes Factors applies logic similar to that used in likelihood ratio tests, except the marginal likelihoods of two models are compared rather than their maximum likelihoods. The marginal likelihood (approximated by the harmonic mean of the maximum likelihoods in BayesTraits when the Markov chain has run for a sufficient number of iterations; see BayesTraits manual) of a model is the integral of the model likelihoods over all values of the model's parameters and over all tree results. Thus, to compare two models, the Bayes Factor ‘test statistic’ is: 2(log[harmonic mean(better model)] – log[harmonic mean(worse model)]; any positive value favours the dependent model, but conventionally, a ratio >2 is taken as ‘positive’ evidence, >5 as ‘strong’ evidence and >10 as ‘very strong’ evidence for support of one model over the other ([42]; www.evolution.rdg.ac.uk).

We initially ran ModelTest ver. 3.7 [43] in Paup* on the *cox1* dataset to determine the best model of evolution for subsequent Bayesian analyses – both the hierarchical Likelihood Ratio Test (hLRT) and Akaike Information Criterion (AIC) returned the HKY model. We then used the program BayesPhylogenies to generate a Bayesian phylogeny estimation over 100,000,000 iterations, sampling every 100,000^th^ tree (Pf  = 100,000) using the HKY model. We reviewed the resulting files in Tracer ver. 1.4.1 [44] to check convergence and proceeded with a tree file (with 10% burn-in discarded) of 900 trees, to BayesTraits analysis.

All our analyses used the sub-program ‘Continuous’ within the software package BayesTraits. We felt that the most appropriate approach was to use the program in MCMC mode (vs. ML mode) on a set of trees generated by Bayesian analyses. Such trees come from the posterior distribution, i.e., they are sampled in proportion to their likelihood given the sequence alignment data and the model of sequence evolution; this means that running BayesTraits in MCMC mode is effectively averaging over the tree estimate or treating it as a nuisance parameter.

As we required rooted, fully-resolved trees, we assigned an arbitrary individual bearing the most common haplotype (see Figure 2) to the outgroup and forced the analysis to retain zero length branches (using the pset collapse = no command in Paup*) rather than collapsing branches. We note that this approach may affect the comparison of Model A and B in BayesTraits as an incorrect root node may hamper the detection of trait evolution, hence we repeated our analyses assigning another arbitrary individual bearing the most common haplotype to the root node and checked for concordance of results.

First, we used BayesTraits (in MCMC mode) to examine the correlation between the root-to-tip distance (i.e., mutation rate) of our phylogenetic estimates and the trait (mass-specific metabolic rate) under both the Random Walk (Model A) and Directional (Model B) models of trait evolution. Initial runs showed that the MCMC chain was not mixing well. This problem was solved by scaling the metabolic rate dataset up by a factor of 1,000, and by lowering the ratedev parameter to 0.002 (Mark Pagel, personal communication). Second, we performed additional analyses under Model A to investigate whether metabolic rate was dependent on the underlying tree. This was assessed by calculating the Bayes Factor for a comparison between a model in which the lambda (λ) parameter was freely estimated versus a model in which it was set to 0 (the latter corresponds to variation in the trait being entirely independent of the underlying phylogeny).

## Results

### Metabolic Rate Analysis

Average metabolic rate among the six populations ranged from 0.0009 – 0.0029 µlO_2_.µg^−1^.hr^−1^ (average: 0.0019±0.0002 µlO_2_.µg^−1^.hr^−1^ (S.E.M.)). Generalized Linear Model (GLM) analyses performed in an additional study [37], showed that metabolic rate differences among populations were significant (*F*_5,42_ = 8.196; *P*<0.001) and a statistical relation to mass was not found (*F*_5,42_ = 0.531; *P* = 0.470).

### Haplotype Network Analysis

**Figure 3:**
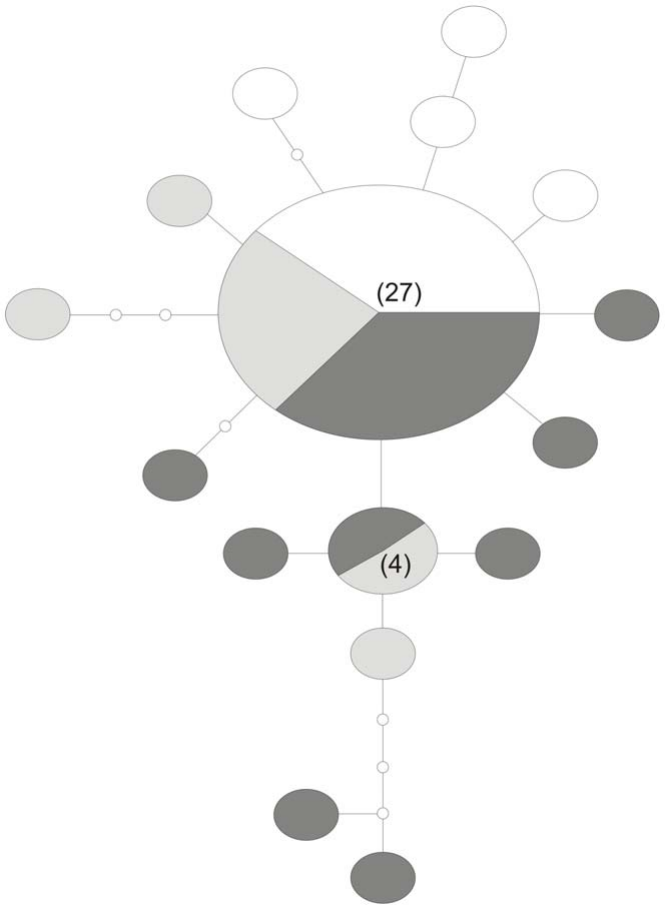
Haplotype network for the mtDNA *cox1* dataset for *Cryptopygus antarcticus travei* from Marion Island. Each large circle represents a unique haplotype and each line represents one mutational step. Numbers in parentheses indicate haplotype frequency (when >1), while ‘
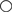
’ indicates an inferred or missing haplotype. Shading indicates whether individuals with that haplotype have low (<0.010 µlO_2_.µg^−1^.hr^−1^; white), medium (0.0010≤*x*≤0.0020 µlO_2_.µg^−1^.hr^−1^; light grey) or high (>0.0020 µlO_2_.µg^−1^.hr^−1^; dark grey) metabolic rates. This figure is not to scale.

There were a total of 16 unique haplotypes for the *cox1* dataset (GenBank Accession No.s: GQ268918-268931) and the maximum number of mutational steps between these was nine (Figure 3). The haplotype network showed a pattern of one most common (i.e., presumed ‘ancestral’) haplotype (n = 27) from which several singletons (and one haplotype with n = 4) were derived. Earlier work [37] showed that haplotype-sharing among *C. a. travei* populations was common, with the number of haplotypes present in each population relatively high and usually including a proportion of unique haplotypes. This work also suggested that populations were genetically differentiated (e.g. ϕ_st_ values, which measure population differentiation, were mostly large and significant).

Of the 45 individuals, 17, 11 and 17 corresponded to ‘low’, ‘medium’ and ‘high’ metabolic rate categories, respectively. The ancestral haplotype had a mixture of individuals with ‘low’, ‘medium’ and ‘high’ metabolic rates, however a visual pattern where more derived haplotypes (i.e., those with a greater number of mutational steps from the proposed ancestral haplotype) have higher metabolic rates was evident in Figure 3. In particular, 11 of the 15 derived haplotypes (i.e., 73.3%) were from individuals with ‘medium’ or ‘high’ metabolic rates. Thus there appears to be some support for an overall pattern of metabolic rate structure coinciding with genetic structure.

### Combined Metabolic Rate and DNA Mutation Rate Analyses

#### Randomisation test analyses

The randomisation test analyses we performed were unable to provide statistical support for relationships among the parameters we tested at a 5% level of significance. Specifically, for the first test statistic we evaluated, *T*_1_(0) (which measures the similarity in metabolic rates of individuals with identical haplotypes), *P* = 0.061, and for *T*_1_(1) (which measures the similarity in metabolic rates of individuals that differ by at most one mutational step in the network in Figure 3), *P* = 0.208. The second test statistic, *T_2_*, measures whether there is a directional change in metabolic rates as we move away from the putative root of the haplotype network (Figure 3) and for this test, *P* = 0.871.

#### BayesTraits analyses

**Figure 4:**
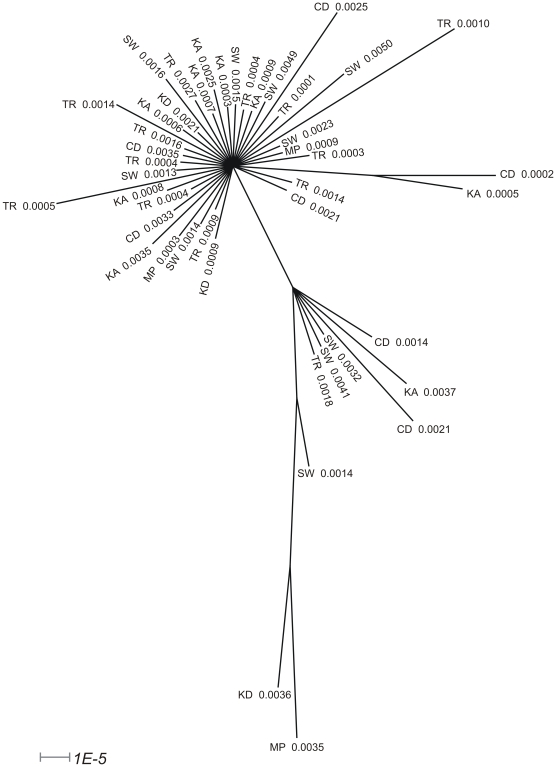
Majority-rule consensus tree of the 900 post-burnin trees from the BayesPhylogenies analysis. Metabolic rates and population codes of individuals are presented at tree tips. Population codes correspond to: CD  =  Cape Davis, KA  =  Katedraal, KD  =  Kildalkey Bay, MP  =  Mixed Pickle, SW  =  Swartkops Point, TR  =  Trypot Beach. Branch lengths (shown by the scale bar in the bottom-left of the figure) are averages over the length of the branch in the trees in which it appeared.

The 900 trees from the post burn-in posterior distribution of the BayesPhylogenies analysis are summarised as a consensus tree in Figure 4. The lack of resolved relationships in Figure 4 (in particular the largest polytomy includes the 27 taxa that form the main group in the haplotype network) is expected given the haplotype network (Figure 3) and highlights the importance of averaging over different possible coalescent histories (i.e., using MCMC mode in BayesTraits). The branch lengths shown are means (i.e., the length of a given branch is the average value of that branch length taken over all the trees it appears in); they indicate that there is variation in the root to tip distances when the root is chosen to be an arbitrary taxon from the main haplotype group (Figure 4).

For the first analysis run in BayesTraits (comparing Model A to Model B), we found that, while Model B had a slightly better marginal likelihood, the evidence to support this was not strong (i.e., Bayes Factor <2) (Table 1). This means our analyses are unable to reject a model in which metabolic rate is not correlated with greater genetic distance to the ancestral node.

**Table 1:** Harmonic mean of log likelihood values of metabolic rate and DNA mutation rate correlation tests.

Model	Harmonic mean of log likelihood values	Bayes Factor
A	−77.4104	0.8476
B	−76.5628	
A(λ = est)	−77.0837	21.9678
A(λ = 0)	−99.0515	

All tests performed in BayesTraits on the springtail *Cryptopygus antarcticus travei* from sub-Antarctic Marion Island.

However, in our subsequent BayesTraits analysis examining the lambda parameter within Model A, we found a Bayes Factor >10 between the model where λ was estimated and the model where λ = 0 (Table 1). This means there is strong evidence that λ≠0, i.e., that metabolic rate is dependent on the underlying mtDNA tree.

## Discussion

Metabolic rate, body size, generation time and effective population size vary through evolutionary time. These changes likely introduce substantial noise to comparisons of physiology and life-history parameters with rate of evolution. Despite this, several researchers have reported observing correlations between rate of evolution and other factors [5], [45], [46]. This includes work that looked at evolutionary rates in two lineages of Hawaiian *Drosophila* species and showed that population-level phenomena can be important in understanding evolution at the molecular level [26], [47]. Metabolic factors were also highlighted as important correlates (if not actual determinants) of variation in evolutionary rates of hummingbirds, whose metabolism may be great enough to alter substitution rates at the level of the nuclear genome [6]. Slow mitochondrial evolution in turtles has been shown to potentially correlate with generation time and/or metabolic rate [23] and further studies supporting the metabolic rate hypothesis include the work on aging in human tissues [21], a rate assessment performed for marine turtles [16], and a study of tube-nosed seabird evolution [12].

In contrast, several studies have failed to find support for the metabolic rate hypothesis [3], [4], [24], [48], or have conversely supported body temperature [3], generation time [2], speciation [49], [50], or some combination of these factors [51]. Most recently, a study on the New Zealand tuatara noted the highest rate of molecular change recorded in a vertebrate, but contrastingly slow tempos of metabolism and growth, and a long generation time for this reptile [52].

The current study is among the first to examine this clearly complex relationship at the intra-specific level, and began with what appeared to be a degree of support for the proposed relationship between metabolic rate and DNA mutation rate. This support was in the graphical form of a haplotype network onto which metabolic rates were mapped. However, the results of our analyses that tested for a direct correlation between these variables while accounting for the underlying phylogeny were unable to strongly corroborate this initially suggestive pattern in *C. a. travei*. In other words, there is not strong evidence that metabolic rate directly increases or decreases with evolutionary distance of individuals from the putative root of the tree. Conversely, subsequent analysis within a BayesTraits lambda framework provided strong evidence (Bayes factor >10) for the hypothesis that metabolic rate is dependent on the underlying tree. This was also weakly supported by the randomisation test statistic *T*_1_(0), which tested if identical haplotypes had more similar metabolic rates than expected by chance, and had a *P*-value of 0.061. Thus, we conclude that, while we find no strong evidence to suggest that metabolic rate and mutation rate (when measured as the root to tip path length) are correlated directly, there is an indirect relationship between these two variables, such that lineages with related mtDNA also have similar metabolic rates.

Testing for a direct correlation between DNA mutation rate and metabolic rate as well as several other life history traits is rendered difficult for several reasons [4]. For example, we cannot assume that relative rates of oxygen radical production in gonad cells are effectively measured by whole body metabolic rate estimates [3], and although evolutionary rates reflect the evolutionary history of each lineage, life history variables are measured in the present time and retain little information about the way they may have changed over time across a lineage [14]. In addition, metabolic rate is clearly not the only causative force leading to DNA damage [34] and a variety of factors are likely to contribute to the likelihood of any mutations becoming fixed in the population (e.g. protein function, purifying selection, population size) [53].

In relation to *C. a. travei* from Marion Island, it is certainly possible that differences among individuals relate to different selective pressures among different populations. In particular, populations from the western side of the island (SW, MP, CD) have higher mean metabolic rates than populations located on the eastern side of the island (KA, KD, TR) (Figure 1), and this may suggest underlying environmental differences among locations. Such variation over sufficient timescales may initiate selection processes in certain loci.

Finally, the mtDNA *cox1* gene may not be ideal for testing these relationships and the sequence lengths employed here (516 bp) may also be insufficient. Indeed, close inspection of the BayesPhylogenies tree file generated in this study revealed that many different coalescent histories are compatible with the data (as expected given the haplotype network; Figure 3), and this could obscure any association between mutation rate and metabolic rate. Unfortunately, this limitation, which essentially relates to a poor phylogenetic signal in our dataset, lies at the heart of the intra-specific approach and is likely to represent the most severe hindrance to studies of this type.

Although we stand by the *a priori* assumption underlying our research (that a functional relationship exists between mtDNA haplotype and metabolic rate based on the relationship between metabolism, oxidative stress and DNA mutation; see Introduction), we must acknowledge the limits of our approach in discriminating between the effects of metabolic rate and other important factors which cause variation in evolutionary rate (i.e., population size and/or selection coefficients operating at the molecular level). With this in mind, we intend our results to be interpreted as a ‘first step’ in the analysis of this complicated issue to expand the metabolic theory of ecology, and hope that they stimulate further studies of mutation rate at the intra-specific level.

In particular, we recommend that future work include further analysis following our methodological approach and using phylogenetically independent contrasts (PICs; see [27]). Closely related sister taxa with differing life history variables such as generation time, body size, colonisation histories, population size, potential selection coefficients and of course, metabolic rates would provide fertile ground for future study of this issue. Examinations at the intra-specific level using larger datasets and including multiple genetic loci (including nuclear genes) are also envisaged as steps to further enlarge our preliminary demonstration. These latter suggestions in particular would aid the process by giving greater power (i.e., phylogenetic signal) to statistical tests such as the ones performed here. Indeed, we strongly recommend that analyses of this type be repeated as larger datasets become available, and expect that recent advances in DNA sequencing technology allowing higher throughput (e.g. of complete mtDNA genomes) are likely to make such studies feasible in the near future. Finally, our work highlights the need for development of new statistical approaches that seek to accommodate coalescent processes which could potentially drive variation in branch lengths independently of any underlying variation in mutation rate. We look forward to such advances and the contribution they may make to the generation of general conclusions concerning sources of rate heterogeneity.
